# Anticholinergic and Sedative Medication Burden in Croatian Older Adults: EuroAgeism Cohort Findings

**DOI:** 10.3390/pharmacy13050144

**Published:** 2025-10-06

**Authors:** Margita Držaić, Iva Bužančić, Ingrid Kummer, Andrea Bošković, Dragan Glavaš, Maja Ortner Hadžiabdić, Jovana Brkić, Daniela Fialová

**Affiliations:** 1 Faculty of Pharmacy and Biochemistry, University of Zagreb, 10000 Zagreb, Croatia; margitadrzaic@gmail.com (M.D.); ibuzancic@gmail.com (I.B.); 2 City Pharmacy Zagreb, 10000 Zagreb, Croatia; 3 Department of Social and Clinical Pharmacy, Faculty of Pharmacy in Hradec Králové, Charles University, 500 03 Hradec Králové, Czech Republicjovanabrkic37@gmail.com (J.B.);; 4 Department of Biomaterials and Biotechnology, University Medical Center Groningen, 9713 AV Groningen, The Netherlands; a.boskovic@umcg.nl; 5 University Department of Psychology, Catholic University of Croatia, 10000 Zagreb, Croatia; dragan.glavas@unicath.hr; 6 Department of Social Pharmacy and Pharmaceutical Legislation, Faculty of Pharmacy, University of Belgrade, 11221 Belgrade, Serbia; 7 Department of Internal Medicine and Geriatrics, 1st Faculty of Medicine in Prague, Charles University, 12108 Prague, Czech Republic

**Keywords:** anticholinergic, sedative, drug burden index, older adults, self-reported health, healthcare utilization

## Abstract

Use of anticholinergic and sedative medications is potentially inappropriate in older adults due to associated adverse effects, including impaired cognitive and physical function. This study evaluated anticholinergic and sedative burden in Croatian community-dwelling older adults using the Drug Burden Index (DBI) and examined its association with self-reported health and healthcare utilization over 12 months. This observational, cross-sectional study, part of the EuroAgeism H2020 ESR 7 project, included conveniently sampled adults ≥ 65 years from community pharmacies in three Croatian regions. Data were collected using a standardized research questionnaire. DBI was used to quantify exposure to anticholinergic and sedative medications. Multivariate regression analyses examined associations between DBI and health outcomes, using logistic regression for binary outcomes and linear regression for self-reported health. Among 388 participants (63.7% female, median age 73), most had multimorbidity (median five diagnoses) and polypharmacy (63.9%), while 57% used at least one DBI medication—most commonly diazepam (15.5%) and tramadol (14.7%). High DBI (≥1) independently predicted more emergency department (ED) visits (OR = 2.45) and worse self-rated health (B = −0.26), but not hospitalization. High DBI in older adults was associated with more ED visits and poorer self-rated health, highlighting the need for targeted interventions to reduce anticholinergic and sedative use in this vulnerable population.

## 1. Introduction

As the world population ages, the number of medications that individuals use increases. Among various medications used by older adults, use of potentially inappropriate medications (PIMs) is considered risky in this vulnerable population group due to age-related physiological changes, risk of multiple comorbidities and polypharmacy [1]. PIMs are considered unsuitable for older patients due to a poor benefit-harm balance and available safer alternatives [2]. Some of the most commonly prescribed medications in older adults are medications with anticholinergic and sedative properties, the use of which is considered potentially inappropriate in this age group [3,4,5]. They have been associated with a wide range of adverse outcomes, i.e., excessive sedation and risk of falls, blurred vision, and dry mouth [6], some of which may limit cognitive and physical functions [7,8,9]. 

So far, a number of tools have been developed to quantify anticholinergic [10] and sedative medication burden [11,12,13]. The Drug Burden Index (DBI) is an example of a validated measure used to assess a person’s total exposure to both, medications with anticholinergic and/or sedative properties [14] and is developed with an intention to be an evidence-based risk assessment tool to guide prescribing in older adults. Its advantage lies in the fact that it uses a pharmacological equation that incorporates both, drug dose and dose–response principles [15,16]. Numerous international studies have confirmed an association between higher DBI score and negative outcomes in older adults, like poorer physical function [15,17,18,19], falls [20,21], frailty [22,23], and mortality [24,25]. It was also found that higher DBI correlates with increased hospitalization [26,27] and difficulties in performing activities of daily living (ADLs) [17,18]. On the other hand, there have been mixed reports regarding the relationship of DBI with mortality [26,28,29,30] and cognitive function [14,31]. Consequently, reducing the use of anticholinergic and sedative medications may have important health benefits in older people. 

In recent decades, the proportion of older adults has been steadily increasing worldwide, reflecting global trends of population aging. Croatia follows a similar pattern: according to the 2011 population census, 17.7% of the population was aged ≥65 years, while this share rose to 22.4% in the 2021 census [32]. With the aging of the population, the risk of potentially inappropriate prescribing increases significantly. Several studies conducted in Croatia have reported a high prevalence of PIMs among older adults, with rates exceeding 60% [33,34]. However, despite the documented high rates of PIM use, the prevalence of anticholinergic and sedative medication use—key contributors to drug-related adverse outcomes—has not yet been investigated in the Croatian population. 

Hence, this study aimed to evaluate, for the first time, anticholinergic and sedative medication burden in Croatian community-residing older adults using the DBI tool. Furthermore, we examined the association between DBI score and patient-related health outcomes which included self-reported health status and healthcare utilization. Healthcare utilization was assessed by analyzing participants’ self-report of any hospitalization and emergency department visits in the previous 12 months.

## 2. Materials and Methods

### 2.1. Study Design

This was an observational, cross-sectional study conducted in the community setting as a part of the EuroAgeism H2020 ESR 7 international project entitled *“Inappropriate prescribing and availability of medication safety and medication management services in older patients in Europe and other countries”.* The study sample included 391 conveniently selected older patients (≥65 years) who attended community pharmacies in three geographically distinct Croatian regions (north-eastern continental Slavonia, north-western City of Zagreb and coastal Istria and Kvarner). All eligible patients were assessed based on inclusion and exclusion criteria. Patients were included in the study if they were in stable health status, meaning no intensive care, no acute worsening of health status requiring hospitalization or emergency department visits in the last three days, and no palliative or terminal care. Criteria to exclude patients from the study were severe dementia (Mini Mental State Examination (MMSE) score below 10) or severe communication disorders (unable to hear or speak). Only patients able and willing to give written informed consent were included in the study. Anonymity was guaranteed by assigning a unique code number to each participant. The study was approved by the Ethical Committees of both the Faculty of Pharmacy in Hradec Králové, Charles University, Czech Republic, and the Faculty of Pharmacy and Biochemistry, University of Zagreb, Croatia.

### 2.2. Data Collection

For the purpose of this analysis, we limited the sample to those patients who used at least one medication in therapy (n = 388). Data were collected using a structured, standardized, and validated research questionnaire developed for the purpose of the EuroAgeism H2020 ESR 7 project in the period from July 2019 until December 2020. Data were obtained from patients’ interviews, the community pharmacy database of dispensed medications, and, if necessary, medical records provided by patients. Data collection, using a comprehensive questionnaire consisting of 17 sections, included information on major sociodemographic (age, gender, education, marital status, and living arrangements) and lifestyle characteristics, nutritional status, clinical diagnoses and symptoms, conducted laboratory tests, medications used in past seven days, healthcare utilization, self-reported health status, occurrence of falls, and pain. Also, a set of comprehensive geriatric assessment (CGA) variables were collected: activities of daily living (ADLs), mobility and strength, frailty score, and cognitive and mood status. Comorbidity scores of the patients were calculated using the age-adjusted Charlson Comorbidity Index (CCI) [35]. Patients were divided into three groups: mild, with a CCI score of 1–2; moderate, with a CCI score of 3–4; and severe, with a CCI score ≥ 5 [36,37,38]. Polypharmacy was defined as concurrent use of five or more drugs [39]. The following geriatric syndromes (GSs) were considered in final analysis: falls, urinary incontinence, constipation, vision impairment, hearing impairment, pressure ulcers, sleeping problems, and dysphagia. 

### 2.3. Outcome Measures

The primary outcome measure was the prevalence of exposure to anticholinergic and/or sedative medications measured by the DBI tool in Croatian community-residing older adults. The secondary outcome measure was the association between DBI score and patient-related health outcomes, including self-reported health status and healthcare utilization, measured by participants’ self-reporting of any hospitalization and emergency department visits in the previous 12 months.

#### 2.3.1. Exposure to Anticholinergic and/or Sedative Medications

Information on medication use was collected during the patients’ interviews and from community pharmacies’ dispensing records. Both data on prescription (Rx) and over-the-counter (OTC) medications used in the past seven days were collected. Details regarding medication name, route of administration, dose, and frequency and duration of consumption were gathered. Medications were categorized according to the Anatomical Therapeutic Chemical (ATC) Classification System recommended by the World Health Organization [40]. The DBI tool was used to quantify the use of medications with sedative and/or anticholinergic properties. The DBI score for each patient was calculated using the following equation:

$$DBI=B_{AC}+B_{s}$$

where B_AC_ indicates the burden from medications with anticholinergic effects and B_S_ that from those with sedative effects. DBI score for each medication was calculated using the following equation:

$$DBI=\sum \frac{D}{D+\delta }$$

where D is the daily dose taken by the patient and δ is the minimum effective daily dose for each medication. The daily dose (D) for individual medication was calculated by multiplying the individual doses and the total number of doses per day. In cases where the medication has been prescribed as needed, a single dose is taken into account when calculating the DBI score, thus obtaining an approximation of medication use as needed, while avoiding complete exclusion of the medication from the analyses. The total DBI score was then recoded into a categorical variable (none (0), low (>0 and <1) and high (≥1)) as in previous studies [41,42,43]. Medications with both anticholinergic and sedative properties were classified as anticholinergic, according to Hilmer et al. [14]. Dietary supplements, medicinal products, and topical medications without significant systemic effects were excluded from DBI calculations as per previous studies [14,42,44].

From several previously published DBI lists [14,41,45,46], we selected the one developed in Ireland as the reference list [45] due to its best match with the medications registered and approved in our country. That list was then adjusted to contain only medications authorized and available in the Republic of Croatia. For this purpose, we used the Basic and Supplementary List of Medicines of the Croatian Health Insurance Fund (CHIF) and the database of medicinal products authorized by the Croatian Agency for Medicinal Products and Medical Devices (HALMED) or approved by a centralized procedure by the European Medicines Agency (EMA). The final list of DBI medications and their minimum effective daily doses is provided in Supplementary Table S1. While the reference DBI list contained a total of 156 medications, the adjusted list included a total of 92 medications, of which 8 had an anticholinergic effect, 61 had a sedative effect, and 23 had both an anticholinergic and sedative effect.

#### 2.3.2. Self-Reported Health Status

Self-reported health status was measured by using a single question “*In general, how would you rate your health?*”. Responses were assessed using a five-point Likert scale ranging between “very poor”, “poor”, “moderate”, “good”, and “very good”. Responses of “very poor” and “poor” were combined because of the low number of responses in the “very poor” category (n = 6), creating a four-category self-reported health variable.

#### 2.3.3. Healthcare Utilization

Healthcare utilization was based on participants’ self-reporting of any hospitalization and any emergency department visit (ED-visit) in the previous 12 months.

### 2.4. Statistical Analysis

Descriptive statistics were used to describe the study population. The normality of distribution of numerical variables was tested using Shapiro–Wilk test. Due to non-normally distributed data, continuous variables were presented as median and interquartile range (IQR). To test the differences in DBI scores between groups, Mann–Whitney’s U test was used for binary variables and Kruskal–Wallis’s test for variables with more than two categories. In this sample, exposure to DBI medications was categorized as none (DBI = 0), low (DBI > 0 and <1), or high (DBI ≥ 1). Multivariate regression analysis was performed to determine associations between categorized DBI score and patient-related health outcomes. For the binary outcomes (hospitalization and ED-visits in previous 12 months), logistic regression was used, with results presented as odds ratios (ORs) with a 95% confidence interval (95% CI). For self-reported health, linear regression was used with results presented as B coefficients with 95% CI. For all analyses, a *p* value < 0.05 was used to indicate statistical significance. Data were analyzed as available, without any imputation for missing data. Collected data were analyzed using IBM SPSS Statistics for Windows, Version 20.0 (IBM Corp., Armonk, NY, USA).

## 3. Results

### 3.1. Sociodemographic and Clinical Characteristics of Study Participants

The final analysis included 388 participants who were predominantly female (n = 247; 63.7%) with the median age of 73 (IQR 69–80). Table 1 shows the main sociodemographic and clinical characteristics of participants. The median number of current diagnoses per participant was 5 (IQR = 3–8), with hypertension (76.1%), dyslipidemia (48.3%), and vision impairment (41.7%) being the most common. Median age-adjusted CCI score was 4 (IQR = 3–5), while severe CCI score was identified in 126 (34%) of the participants. The most prevalent geriatric syndromes were vision impairment (41.7%), sleeping problems (33.8%), and hearing impairment (23.6%). The median number of used medications (Rx + OTC) per participant was 6 (IQR = 4–8). Polypharmacy (5 + medications) was identified in 248 (63.9%) and excessive polypharmacy (10 + medications) in 43 (11.1%) participants. The most commonly prescribed medications according to ATC code were those for the cardiovascular system (37.93%), alimentary tract and metabolism (16.86%), and nervous system (16.02%).

Hospitalization in previous 12 months was reported in 51 (13.1%) of participants, where 8 (2.1%) of them were hospitalized in the last two weeks prior to data collection. Most of the participants, 30 (7.9%) of them, were hospitalized only once in the previous 12 months. At least one ED-visit in the previous 12 months was reported in 97 (25.1%) of participants. On average they had 1.49 ± 1.081 ED-visits, with one participant having 10 of them in the previous 12 months. Most participants, 151 of them (39.0%), self-reported their health as being moderate. A detailed overview of self-reported health, hospitalizations, and ED-visits in the previous 12 months is shown in Table 1. 

### 3.2. Prevalence of Exposure to DBI Medications

Overall, 221 (57.0%) of participants were using at least one DBI medication. Mean number of DBI medications per participant was 0.92 ± 1.08 (median (IQR) = 1.00 (0.00–1.00)), with 7 being the maximum number of DBI medications per patient. Low DBI score (>0 and <1) was found in 39.4% of the participants and a high DBI score (≥1) in 17.5% of them. The mean DBI score per participant was 0.51 ± 0.65 (median (IQR) = 0.27 (0.00–0.83)). Details of DBI exposure are shown in Table 1. The total number of used DBI medications was 351. Of those, the most commonly used drug class was benzodiazepines (144; 37.11%), followed by opioids (64; 16.49%) and antidepressants and benzodiazepine-related drugs (31; 7.99%). Diazepam and tramadol were the most frequently identified DBI medications (15.46% and 14.69%, respectively) (Table 2). A detailed overview of specific DBI medications identified in the sample and their prevalences are presented in Appendix A.

### 3.3. Factors Significantly Associated with DBI Score

Our results have shown that DBI score was statistically significantly higher in females than in males. A statistically significant difference in DBI score was also observed in participants from different regions, with those living in the eastern part of Croatia (Slavonia) having a higher DBI score. Participants who used five or more medications and had more current diagnoses had a significantly higher DBI score. Detailed results are summarized in Table 3. 

### 3.4. Results of Multivariate Regression Analysis

Binary logistic and linear regression models were used to identify potential predictors for patient-related health outcomes (self-reported health and healthcare utilization). The independent variables of DBI score, age, gender, polypharmacy, and number of current diagnoses were included in the multivariate regression model. Results are summarized in Table 4. High DBI score (DBI score ≥ 1) vs. none was significantly associated with more ED-visits in the previous 12 months (OR 2.446, 95% CI 1.216, 4.917, *p* = 0.012) and poorer self-reported health (B = −0.262, 95% CI −0.503, 0.021, *p* = 0.033). There was no significant association between any category of DBI score and hospitalization in the previous 12 months (*p* > 0.05). Except for a high DBI score, a significant independent predictor of ED-visits in the previous 12 months was also the number of current diagnoses (OR 1.090, 95% CI 1.005, 1.181, *p* = 0.033). For self-reported health, except for a high DBI score, significant predictors were also gender (B = −0.176, 95% CI −0.339, −0.013, *p* = 0.035) and number of current diagnoses (B = −0.087, 95% CI −0.115, −0.059, *p* < 0.001). Out of all the independent predictors for hospitalization in the previous 12 months, only gender (OR 0.436, 95% CI 0.236, 0.806, *p* = 0.008) and the number of current diagnoses (OR 1.111, 95% CI 1.009, 1.223, *p* = 0.033) were found to be significant.

## 4. Discussion

The prevalence of anticholinergic and sedative drug use among community-dwelling older adults in Croatia mirrors those in similar populations in neighboring countries but is higher when compared to western or northern European countries or available data from other countries worldwide [47,48,49,50,51,52]. Croatian community-dwelling older adults are exposed to a higher anticholinergic drug burden than their peers in the Netherlands, Germany, Turkey, or the USA, which, based on our results, is primarily due to a higher sedative burden [52,53,54,55]. 

Benzodiazepines were the most commonly used DBI medications, underscoring long-standing concerns about inappropriate prescribing in older populations. Diazepam and tramadol were particularly prevalent, despite being recognized as PIMs due to their sedative burden and associated risks (e.g., falls, cognitive decline) [14,56].

Gender and regional differences were also observed. Women exhibited higher DBI scores than men, a finding consistent with earlier studies suggesting gender disparities in both prescribing patterns and healthcare-seeking behavior [57]. Moreover, participants from eastern Croatia (Slavonia) had higher DBI scores compared to those from more urbanized areas such as Zagreb or the coastal Istria and Kvarner region. These findings may reflect regional differences in prescribing culture, access to healthcare services, or socioeconomic determinants that warrant further qualitative exploration.

Beyond examining the prevalence and sociodemographic patterns of higher DBI prescribing, it is essential to explore the association between anticholinergic and sedative exposure and health-related outcomes. Our findings align with the previous literature that has linked higher DBI scores with adverse clinical outcomes, including impaired physical function, frailty, cognitive decline, and institutionalization [27,58,59,60,61]. This study expands the evidence by demonstrating that even in relatively functional, community-dwelling older adults, DBI exposure remains prevalent and clinically consequential.

Inappropriate use of anticholinergic and sedative medications has been linked to a number of unwanted and undesirable patient-related outcomes across different health domains [27,58,59]. For this older adult population, higher anticholinergic burden was associated with higher healthcare utilization and lower self-reported health status. Increased healthcare utilization implies deterioration in patients’ health. Deterioration can further decrease functional capacity, lead to increases in unwelcome polypharmacy exposure, and negatively impact self-reported health status. Additionally, increased healthcare utilization often triggers higher health system costs. Similar findings are reported in studies by Jaggi et al. and Chatterjee et al., stating increased costs and healthcare resource utilization among patients with any anticholinergic exposure [60,61]. It is important to reflect on the effect of anticholinergic drug burden on self-reported health status. As a multidimensional construct, self-reported health status shows a strong association with physical function and can reliably be used by healthcare providers as a part of overall health assessment [62]. Adverse side effects resulting from use of anticholinergic medications, such as daytime sedation, blurred vision, cognitive decline, or urinary retention, can impair older adults’ sense of physical and mental well-being and result in a more negative valuation of overall health and quality of life. Healthcare providers should pay special attention to those with changes in self-perceived health status and concomitant use of anticholinergic and/or sedative medications. Research supports this notion, with findings expressing anticholinergic burden’s association with both long- and short-term poorer physical and mental function and decline in quality of life [58,63,64]. 

Higher DBI scores were found in older adults who experienced falls in the last twelve months, underscoring the known impact of anticholinergic and/or sedative medications on balance, walking speed, and mobility [58]. Use of drugs with anticholinergic properties was linked with impaired cognition and mobility, especially in old-old adults (>75 years of age). These medications can affect executive functions and additionally contribute to falls [65]. Increased risk or experience of falls can lead to further health decline and mortality [66]. Furthermore, participants using five or more medications and those with a greater number of current diagnoses had significantly higher DBI scores, suggesting that multimorbidity and subsequent polypharmacy are key contributors to increased anticholinergic exposure. These findings align with those of Ulugerger Avci et al., who reported that polypharmacy and higher comorbidity levels were significantly associated with increased anticholinergic burden, or those of Sürmeli et al., who stated that polypharmacy and frailty are significant predictors of high anticholinergic burden [52,67].

The results emphasize that anticholinergic burden both impacts and is influenced by multiple health factors. This can create a cycle that can adversely affect safety, functional independence, and overall well-being in older adults. Moreover, findings indicate that vigilance is needed when prescribing and dispensing medication with anticholinergic and/or sedative potential to older adults, as well as that timely and continuous assessment of patients’ medication is necessary as they age or their comorbidities progress. Various tools are accessible to healthcare providers which can help in making appropriate prescribing choices, assessment of anticholinergic and/or sedative burden, or deprescribing of these medications [68,69]. Heterogeneity of available evidence, practical challenges in regular clinical use, and lack of inclusion of certain populations, such as considerably frail or cognitively impaired, can hinder both tool utilization and comprehensive care for older adults [70]. While there is a lack of evidence on the success of interventions to reduce anticholinergic burden due to short follow-up time and lacking support on deprescribing, healthcare providers should continue to strive in suggesting and providing deprescribing or other medication optimization services to such patients [71,72]. For certain types of medicines with anticholinergic and sedative properties, such as benzodiazepines and opioid analgesics, the decision of continuing versus discontinuing is supported with more evidence which can help clinicians in day-to-day decision making [73,74,75]. Pharmacist-led geriatric assessment including a deprescribing-focused medication review has shown that a considerable proportion of older adults using benzodiazepines and opioids, medicines comprising a sizable portion of anticholinergic drug list, are candidates for deprescribing [76], further accentuating the need for changes in prescribing practices and patient care. Integrating tools to better assess anticholinergic burden into electronic medical records or healthcare software can facilitate the provision of deprescribing [77].

### Strengths and Limitations

To allow for appropriate interpretation of results it is important to consider potential limitations of this research. The use of the DBI to assess anticholinergic and sedative burden may limit comparability with studies using other anticholinergic-specific scales such as the Anticholinergic Cognitive Burden (ACB) or Anticholinergic Drug Scale (ADS). Unlike these tools, the DBI considers both the drug dose and cumulative burden of both anticholinergic and sedative medications but may not account for all medications with anticholinergic properties. A cross-sectional design prevents confirmation of causality and further longitudinal studies are needed, both for Croatian and other European healthcare systems. Furthermore, future research could focus on interventional studies exploring efficient and successful strategies to mitigate anticholinergic burden, which can then be implemented in healthcare systems. Selection bias might have been introduced as patients were recruited via a convenient sampling method, which makes it difficult to generalize findings on other populations of older adults. This research team was not able to use the entire sample from all included countries in the EuroAgeism H2020ESR 7 project, and analysis was available only for the Croatian cohort. The 17-part questionnaire gathered extensive information on the participant, allowing for a comprehensive evaluation of their health status, though there could be other important patient-related characteristics associated with anticholinergic drug use not collected. The use of additional objective measures of healthcare utilization or health-related characteristics to confirm patient-reported data could lessen potential recall bias. Defining polypharmacy solely by the number of medications has limitations, as it does not account for the patient’s comorbidities and makes it difficult to evaluate the safety and appropriateness of the medication regimen which is necessary for a more comprehensive understanding of a patient’s medication use. Nevertheless, the sample adequately represents Croatian older adults, both in numbers and important clinical characteristics, such as exposure to polypharmacy or use of potentially inappropriate medications [78]. Additionally, this is, to our knowledge, the first study to explore the prevalence of anticholinergic drug use, as well as the association of anticholinergic drug use and patient-related health outcomes for Croatian older adults, highlighting the importance of quantifying the exposure to medication which can negatively affect patient care. 

## 5. Conclusions

The findings underscore the widespread exposure to anticholinergic and sedative medications among community-dwelling older adults in Croatia and its association with negative health outcomes and healthcare utilization. Findings align with international data, reinforcing the role of polypharmacy and multimorbidity as key contributors to higher anticholinergic exposure. Given its multidimensional impact on health and well-being, anticholinergic burden should be routinely assessed and minimized where possible. DBI score is a useful and sensitive measure for identifying older patients at risk and can inform clinical decision making aimed at reducing polypharmacy-related harm. Future longitudinal studies are warranted to explore causal pathways and evaluate the effectiveness of deprescribing interventions in this population.

## Figures and Tables

**Table 1:** Main sociodemographic and clinical characteristics of study population.

Characteristic	n (%) Participants
**Gender**	
male	141 (36.3)
female	247 (63.7)
**Age**	
median (IQR)	73.0 (69.0–80.0)
65–74	215 (56.1)
75–84	134 (35.0)
≥85	34 (8.9)
**Region**	
Istria and Kvarner	119 (30.7)
City of Zagreb	144 (37.1)
Slavonia	125 (32.2)
**Number of current diagnoses**	
≤2	61 (15.7)
3–5	157 (40.5)
≥6	170 (43.8)
**CCI score**	
mean (SD)	4.17 (1.62)
median (IQR)	4 (3.0–5.0)
**CCI score group**	
mild (1–2)	46 (12.4)
moderate (3–4)	199 (53.6)
severe (≥5)	126 (34.0)
**Most common current diagnoses**	
hypertension	293 (76.1)
dyslipidemia	186 (48.3)
vision impairment	161 (41.7)
sleeping problem	130 (33.8)
diabetes mellitus 2	98 (25.5)
**Number of used medications (Rx + OTC) ^a^**	
1–4 medications	140 (36.1)
≥5 medications	248 (63.9)
≥10 medications	43 (11.1)
**DBI score**	
mean (SD)	0.51 (0.65)
median (IQR)	0.27 (0.0–0.8)
**DBI score group**	
0	167 (43.0)
>0 to <1	153 (39.4)
≥1	68 (17.5)
**Geriatric Syndromes (GSs)**	
falls	70 (18.2%)
urinary incontinence	51 (13.3%)
constipation	34 (8.8%)
vision impairment	161 (41.7%)
hearing impairment	91 (23.6%)
pressure ulcers	7 (1.8%)
insomnia	130 (33.8%)
dysphagia	26 (6.8%)
**Current score of frailty test ^b^**	
very fit	45 (11.7)
well	63 (16.4)
managing well	177 (46.1)
vulnerable	65 (16.9)
mildly frail	18 (4.7)
moderately frail	10 (2.6)
severely frail	3 (0.8)
very severely frail	2 (0.5)
terminally ill	1 (0.3)
**Mobility and strength**	
goes out	367 (94.6)
difficulty walking across a room	100 (26.0)
difficulty transferring from a chair	122 (31.8)
difficulty climbing a flight of 10 stairs	195 (50.7)
difficulty lifting or carrying around 5 kg	190 (49.4)
**Hospitalization in previous 12 months**	
yes	51 (13.1)
no	337 (86.9)
**ED-visit in previous 12 months**	
yes	97 (25.1)
no	290 (74.9)
**Self-reported health**	
very poor and poor	42 (10.9)
moderate	151 (39.0)
good	142 (36.7)
very good	52 (13.4)

DBI, *Drug Burden Index*; CCI, *Charlson Comorbidity Index*; ED-visits, *emergency department visits.* ^a^ None of the participants used zero (0) medications. ^b^ Participants being very fit, well, or managing well on the frailty test were considered as not being frail.

**Table 2:** Most frequently used DBI medications in study population.

Medication	Anticholinergic/ Sedative Effect	n (%) Participants
Diazepam	sedative	60 (15.46)
Tramadol	sedative	57 (14.69)
Alprazolam	sedative	53 (13.66)
Zolpidem	sedative	31 (7.99)
Moxonidine	sedative	22 (5.67)
Oxazepam	sedative	16 (4.12)
Escitalopram	sedative	12 (3.09)
Nitrazepam	sedative	10 (2.58)
Loratadine	sedative	10 (2.58)
Sertraline	sedative	7 (1.80)
Duloxetine	sedative	6 (1.55)

**Table 3:** Patient characteristics and health determinants associated with the DBI score.

Variable	Mean DBI Score ± SD	*p* Value	Median DBI Score	IQR
**Gender**				
Male	0.43 ± 0.607	0.035 *	0.00	0.00–0.75
Female	0.56 ± 0.664		0.50	0.00–0.83
**Age group**				
Early age (65–74)	0.51 ± 0.695	0.264	0.27	0.00–0.77
Middle age (75–84)	0.48 ± 0.561		0.27	0.00–0.83
Oldest age (≥85)	0.65 ± 0.656		0.58	0.00–0.96
**Region**				
City of Zagreb	0.50 ± 0.653	0.007 *	0.13	0.00–0.83
Slavonia	0.62 ± 0.656		0.50	0.00–0.91
Istria and Kvarner	0.41 ± 0.613		0.16	0.00–0.67
**Number of current diagnoses**				
≤2 diagnoses	0.14 ± 0.266	<0.001 *	0.00	0.00–0.14
3–5 diagnoses	0.44 ± 0.574		0.27	0.00–0.67
≥6 diagnoses	0.71 ± 0.727		0.66	0.00–1.12
**CCI score group**				
Mild (1–2)	0.49 ± 0.720	0.162	0.14	0.00–0.75
Moderate (3–4)	0.47 ± 0.616		0.27	0.00–0.77
Severe (≥5)	0.60 ± 0.671		0.50	0.00–1.04
**Number of used medications (Rx + OTC)**				
1–4 medications	0.20 ± 0.320	<0.001 *	0.00	0.00–0.50
≥5 medications	0.69 ± 0.713		0.66	0.00–1.00

DBI, *Drug Burden Index*; ED-visits, *emergency department visits*. * *p* < 0.05 is considered statistically significant. Non-parametric tests were used; Mann–Whitney’s U test for binary variables and Kruskal–Wallis’s test for variables with more than two categories (age, region, self-reported health, and number of current diagnoses).

**Table 4:** Multivariate regression analysis showing potential predictors for patient-related health outcomes.

Predictor	ED Visits ^a^		Hospitalization ^b^		Self-Reported Health
	B	OR (95% CI)	B	OR (95% CI)	B (95% CI)
**DBI score**					
None ^#^	1	1	1	1	1
Low (>0 to <1)	0.303	1.354 (0.772, 2.376)	0.565	1.759 (0.854, 3.626)	−0.151 (−0.329, 0.027)
High (≥1)	0.894 *	2.446 * (1.216, 4.917)	0.516	1.675 (0.679, 4.133)	−0.262 * (−0.503, 0.021)
**Age**	0.010	1.010 (0.975, 1.047)	0.011	1.011 (0.967, 1.058)	−0.010 (−0.022, 0.002)
**Gender**					
Male ^#^	1	1	1	1	1
Female	0.161	1.175 (0.708, 1.951)	−0.830 *	0.436 * (0.236, 0.806)	−0.176 * (−0.339, −0.013)
**Polypharmacy**					
No ^#^	1	1	1	1	1
Yes	−0.135	0.874 (0.480, 1.590)	−0.100	0.905 (0.418, 1.957)	−0.086 (−0.275, 0.104)
**No. of** **current** **diagnoses**	0.086 *	1.090 * (1.005, 1.181)	0.105 *	1.111 * (1.009, 1.223)	−0.087 * (−0.115, −0.059)

DBI, *Drug Burden Index*; ED-visits, *emergency department visits*; OR, *odds ratio*; CI, *confidence interval*. * *p* < 0.05 is considered statistically significant; # reference category. ^a^ Overall model fit (χ2(6) = 17.784, *p* = 0.007). The model explained 6.7% (Nagelkerke R^2^) of the variance in ED-visits in the previous 12 months and correctly classified 74.3% of cases. ^b^ Overall model fit (χ2(6) = 15.784, *p* = 0.015). The model explained 7.4% (Nagelkerke R^2^) of the variance in hospitalization in the previous 12 months and correctly classified 86.7% of cases.

## Data Availability

The data presented in this study are available on request from the corresponding author.

## Supplementary Materials

The following supporting information can be downloaded at: https://www.mdpi.com/article/10.3390/pharmacy13050144/s1, Table S1: List of anticholinergic and sedative medications included in the calculation of Drug Burden Index (DBI).

## Appendix A. Prevalence of DBI Medications per ATC Classification

**MEDICATION CLASS**	**MEDICATION**	**WHO ATC CODE/S**	**N**	**% of Participants**
**BENZODIAZEPINES**			**144**	**37.11**
	diazepam	N05BA01	60	15.46
	alprazolam	N05BA12	53	13.66
	oxazepam	N05BA04	16	4.12
	nitrazepam	N05CD02	10	2.58
	lorazepam	N05BA06	5	1.29
**OPIOIDS**			**64**	**16.49**
	tramadol	N02AX02 N02AJ13 ^b^ N02AJ14 ^b^	57	14.69
	codeine	R05DA04 N02BE51 ^c^	4	1.03
	tapentadol	N02AX06	2	0.52
	oxycodone	N02AA05 N02AA55 ^d^	1	0.26
**BENZODIAZEPINE-RELATED DRUGS**			**31**	**7.99**
	zolpidem	N05CF02	31	7.99
**CENTRALLY-ACTING ANTIHYPERTENSIVES**			**22**	**5.67**
	moxonidine	C02AC05	22	5.67
**ANTIDEPRESSANTS**			**31**	**7.99**
	escitalopram	N06AB10	12	3.09
	sertraline	N06AB06	7	1.80
	duloxetine	N06AX21	6	1.55
	mirtazapine	N06AX11	3	0.77
	fluvoxamine	N06AB08	1	0.26
	paroxetine	N06AB05	1	0.26
	trazodone	N06AX05	1	0.26
**ANTIHISTAMINES**			**15**	**3.87**
	loratadine	R06AX13	10	2.58
	fexofenadine	R06AX26	3	0.77
	cetirizine	R06AE07	1	0.26
	levocetirizine	R06AE09	1	0.26
**ANTIEPILEPTICS**			**9**	**2.32**
	carbamazepine	N03AF01	4	1.03
	lamotrigine	N03AX09	2	0.52
	pregabalin	N03AX16	2	0.52
	clonazepam	N03AE01	1	0.26
**ALPHA-BLOCKERS USED AS ANTIHYPERTENSIVES**			**4**	**1.03**
	doxazosin	C02CA04	4	1.03
**PROPULSIVES**			**4**	**1.03**
	metoclopramide	A03FA01	4	1.03
**MEDICATIONS FOR OVERACTIVE BLADDER AND URGE INCONTINENCE**			**10**	**2.58**
	trospium	G04BD09	4	1.03
	solifenacin	G04BD08 G04CA53 ^e^	3	0.77
	darifenacin	G04BD10	2	0.52
	propiverine	G04BD06	1	0.26
**ANTIPSYCHOTICS**			**10**	**2.58**
	promazine	N05AA03	3	0.77
	quetiapine	N05AH04	2	0.52
	sulpiride	N05AL01	2	0.52
	aripiprazole	N05AX12	1	0.26
	haloperidol	N05AD01	1	0.26
	olanzapine	N05AH03	1	0.26
**ANTIPARKINSONICS**			5	1.29
	amantadine	N04BB01	2	0.52
	ropinirole	N04BC04	2	0.52
	pramipexole	N04BC05	1	0.26
**VERTIGO MEDICATIONS**			1	0.26
	cinnarizine	N07CA02 N07CA52 ^f^	1	0.26
**ANTI-DEMENTIA MEDICATIONS**			**1**	**0.26**
	memantine	N06DX01	1	0.26
^a^ Anatomical Therapeutic Chemical classification system, ^b^ N02AJ13-tramadol+paracetamol; N02AJ14-tramadol+dexketoprofen, ^c^ N02BE51-codeine+paracetamol+caffeine+propyphenazone, ^d^ N02AA55-oxycodone+naloxone, ^e^ G04CA53-solifenacin+tamsulosin ^f^ N07CA52-cinnarizine+dimenhydrinate.				
