# Supplementary material for: Anticholinergic and Sedative Medication Burden in Croatian Older Adults: EuroAgeism Cohort Findings
Source: Pharmacy (Basel). 2025 Oct 6;13(5):144. doi: 10.3390/pharmacy13050144 (PMC12567140; doi:10.3390/pharmacy13050144)
Supplement: Supplementary file 1 [file pharmacy-13-00144-s001.zip › pharmacy-3753196-supplementary.pdf]

DRUG	WHO ATC CODE/S	ANTICHOLINERGIC EFFECTS (A)	SEDATIVE EFFECTS (S)	MINIMUM EFFECTIVE DOSE BY ROUTE OF ADMINISTRATION (mg)					
				oral	parenteral	Sublingual/ buccal	transdermal	rectal	inhalation
alprazolam	N05BA12		S	0,5					
amantadine	N04BB01	AC		100					
amisulpride	N05AL05		S	50					
amitriptyline	N06AA09	AC	S	10					
aripiprazole	N05AX12		S	10	10				
asenapine	N05AH05		S			10			
baclofen	M03BX01		S	30	30				
biperiden	N04AA02	AC	S	1					
buprenorphine	N02AE01		S			0,4	0,12		
carbamazepine	N03AF01	AC	S	400				500	
cetirizine	R06AE07		S	10					
chlorphenamine[a]	R06AB04	AC	S	8	3				
cinnarizine	N07CA02<br>N07CA5	AC	S	60					
citalopram	N06AB04		S	10					
clonazepam	N03AE01		S	0,5	0,5				
clozapine	N05AH02	AC	S	25					
codeine	R05DA04<br>N02AJ06		S	120					
darifenacin	G04BD10	AC		7,5					
diazepam[b]	N05BA01		S	1	1			1	
dimenhydrinate	R06AA02<br>N07CA52	AC	S	150					

diphenhydramine	N02BE71<br>N02BE51<br>R06AA02<br>R06AA52	AC	S	50					
doxazosin	C02CA04		S	2					
doxylamine	R06AA09<br>R06AA59<br>N02BE51	AC	S	25					
duloxetine	N06AX21		S	30					
escitalopram	N06AB10		S	5					
fentanyl	N02AB03		S				0,3		
fesoterodine	G04BD11	AC		4					
fexofenadine	R06AX26		S	120					
fluoxetine	N06AB03		S	20					
fluphenazine	N05AB02	AC	S		0,36				
flurazepam	N05CD01		S	15					
fluvoxamine	N06AB08		S	100					
gabapentin	N03AX12		S	900					
glycopyrronium	A03AB02	AC	S	2	0,2				
haloperidol	N05AD01		S	0,5	0,25				
hyoscine butylbromide	A03BB01	AC		30	30				
lamotrigine	N03AX09		S	100<br>200*					
levetiracetam	N03AX14		S	1000					
levocetirizine	R06AE09		S	5					
levomepromazine	N05AA02	AC	S	37,5					
loratadine	R06AX13		S	10					

lorazepam	N05BA06		S	0,5	0,5				
loxapine	N05AH01	AC	S						4,5
memantine	N06DX01		S	20					
methadone	N07BC02		S	5					
metoclopramide	A03FA01<br>N02BE51		S	15					
mirtazepine	N06AX11		S	15					
morphine	N02AA01<br>N02AA51		S	20	6,7				
moxonidine	C02AC05		S	0,2					
nefopam	N02BG06	AC	S						
nitrazepam	N05CD02		S	2,5					
olanzapine	N05AH03	AC	S	5	5				
oxazepam	N05BA04		S	15					
oxcarbazepine	N03AF02		S	600					
oxybutynin	G04BD04	AC	S	5			1,95		
oxycodone	N02AA05<br>N02AA55		S	20	10			20	
paliperidone	N05AX13		S	3	0,89				
paroxetine	N06AB05	AC	S	20					
perampanel	N03AX22		S	4					
phenobarbital	N03AA02		S	60	60				
pramipexole	N04BC05		S	0,088					
pregabalin	N03AX16		S	150					
promazine	N05AA03	AC	S	100					
propiverine	G04BD06	AC	S	15					
quetiapine	N05AH04	AC	S	50					

risperidone	N05AX08		S	1	0,7				
ropinirole	N04BC04		S	2					
rotigotine	N04BC09		S				1		
rufinamide	N03AF03		S	400					
sertraline	N06AB06		S	50					
solifenacin	G04BD08<br>G04CA53	AC		5					
sulpiride	N05AL01	AC	S	400					
tapentadol	N02AX06		S	100					
tizanidine	M03BX02	AC	S	6					
topiramate	N03AX11		S	50					
tramadol	N02AX02<br>N02AJ13		S	200	200				
trazodone	N06AX05		S	100					
trospium	G04BD09	AC		40					
valproic acid	N03AG01		S	1000					
venlafaxine	N06AX16		S	75					
vigabatrin	N03AG04		S	2000					
ziprasidone	N05AE04		S	40					
zolpidem	N05CF02		S	5					
zonisamide	N03AX15		S	300					
atropine[c]	A03BA01<br>A03CB03	AC			0,3				
clobazam[c]	N05BA09		S	10					
clomethiazole[c]	N05CM02<br>N05CX04		S	192					
clomipramine[c]	N06AA04	AC	S	30					

methyldopa[c]	C02AB01		S	500					
pethidine[c]	N02AB02		S		150				
phenytoin[c]	N03AB02		S	200	200				
primidone[c]	N03AA03		S	750					
trihexyphenidyl[c]	N04AA01	AC		5					

WHO ATC, *World Health Organisation Anatomical Therapeutic Classification*

*Minimum effective daily dose if taken concurrently with agents that induce hepatic enzymes including phenytoin, carbamazepine, phenobarbitone, primidone, rifampicin and lopinavir/ritonavir

[a] in Croatia registered only as OTC medication in combination (ATC: N02BE51)

[b] for rectal use registered in Croatia as generic medication

[c] not registered in Croatia, but on a Main List of Medicines of Croatian Health Insurance Fund as generic medication

Sources:

- Main List of Medicines of Croatian Health Insurance Fund (from 15.06.2020.)
- Supplementary List of Medicines of Croatian Health Insurance Fund (from 15.06.2020)
- Database of medicinal products authorized by the Croatian Agency for Medicinal Products and Medical Devices (HALMED) https://www.halmed.hr/Lijekovi/Baza-lijekova/
